# Enhancement of a one-step membrane technique for the treatment of large bone defects by pre-seeding the membrane with CD8 lymphocyte depleted bone marrow mononuclear cells in a rat femoral defect model

**DOI:** 10.3389/fimmu.2024.1488611

**Published:** 2024-10-23

**Authors:** Marissa Penna-Martinez, Andreas Kammerer, Pia Stützle, Sabatian Fees, Savina Behr, Inna Schaible, Katrin Schröder, René Danilo Verboket, Jonas Neijhoft, Ingo Marzi, Christoph Nau, Dirk Henrich

**Affiliations:** ^1^ Department of Trauma Surgery and Orthopedics, University Hospital, Goethe University Frankfurt, Frankfurt am Main, Germany; ^2^ Vascular Research Center, Goethe University Frankfurt, Frankfurt am Main, Germany

**Keywords:** bone marrow-derived mononuclear cells, BMC, CD8 lymphocytes, bone healing, one-step membrane technique, human acellular dermal matrix

## Abstract

**Background:**

The one-step membrane technique, using a human acellular dermal matrix (hADM), is an experimental method for treating large bone defects. This eliminates the need for the Masquelet membrane induction step, shortening the procedure while maintaining effectiveness. However, previous studies showed that colonizing hADM with bone marrow mononuclear cells (BMC) worsens healing, likely due to the presence of CD8+ lymphocytes, which negatively affect bone regeneration. This study aims to investigate whether the negative impact of BMC on bone healing in this technique is due to the CD8+ cell population.

**Materials and methods:**

A 5 mm femoral defect was created in 25 male Sprague-Dawley rats, divided into three groups (G1-G3). BMC were isolated from syngenic donor rats, with CD8+ lymphocytes removed magnetically from the BMC fraction in one group. The defects were filled with bone chips and wrapped with differently treated hADM: G1 received native hADM, G2 received hADM+BMC, and G3 received hADM+BMC-CD8. After 8 weeks, the femurs were evaluated through radiological, biomechanical, and histological examinations.

**Results:**

Bone defects and bone mineral density (BMD) were significantly improved in G3 (hADM+BMC-CD8) compared to G2 (hADM+BMC). Bone volume, bone formation, and median bending stiffness were higher in G3. Immunohistological analysis showed a significant decrease in CD8 cell count in G3, with a lower percentage of IFNγ-producing cells compared to G2.

**Conclusion:**

Depleting CD8+ cells from BMC before colonizing hADM significantly improved bone healing, likely due to changes in the local mediator environment. This suggests that preoperative colonization with CD8+-depleted BMC could enhance the one-step membrane technique.

## Introduction

The surgical treatment of long bone defects caused by high-impact trauma or following debridement of osteomyelitis and pseudarthrosis remains a major challenge for modern trauma surgery and orthopedics. While smaller defects up to 4 cm in length can be treated with corticospongiosal autologous grafts (1, 2), larger defects require complex reconstruction, such as vascularized bone grafting (3, 4), the induced membrane concept (5, 6) or bone transport using an Ilizarov fixator (7). The use of bone graft substitutes has also increased in recent years to reduce the need for autologous bone (2, 8).

The induced membrane concept described by Masquelet and colleagues for the reconstruction of long bone defects is a two-stage surgical technique (6, 9). In the first operation, a PMMA (polymethyl methacrylate) spacer is inserted into the bone defect, which induces the formation of a membrane. After 8-12 weeks, the PMMA spacer is removed in a second operation and the induced membrane tube is filled with iliac crest cancellous bone or RIA (reamer-irrigator-aspirator) cancellous bone. Over the course of several months, complete bone healing occurs with full bone function. The concept of the induced membrane is now widely used and has been published in numerous clinical case reports in recent years (10–14).

The lengthy two-stage Masquelet procedure could be shortened by replacing the induced membrane with an artificial membrane, thus eliminating the first surgical procedure for membrane induction. Own recent work in the rat femoral defect model showed that the use of an human acellular dermal matrix (hADM), which has a similar structural composition to the induced membrane, can achieve a good bone healing result comparable to the two-stage Masquelet technique, if vital spongious bone is used as defect filling (15).

Since the artificial membrane, in contrast to the induced membrane, is neither initially vascularized nor cellularized, nor does it secrete regeneration-promoting growth factors, and can only influence the healing process via its barrier effect, we attempted to further increase the effectiveness of the hADM by colonizing it with bone marrow mononuclear cells (BMC). Bone-marrow mononuclear cells have been shown to be strong inducers of bone healing. After implantation of β-tricalcium phosphate (β-TCP) scaffolds colonized with BMC into a large rat femoral defect, bony consolidation was shown to be comparable to the use of autologous bone (16–21).

However, recent analyses indicate that the BMC colonized hADM does not lead to an improvement in the bone healing result in the rat femoral defect model, and in some cases even to a reduction in new bone formation (22, 23). This surprising finding could be due to the cellular composition of the BMC. BMC typically contain 2-3% hematopoietic stem cells (CD34+), 0.02% MSC, and approx. 8-14% monocytes, which are also essential for a bone regenerative effect (21, 24). The remaining cells consist of, among others, approximately 30% lymphocytes. The mean percentage of CD8 cytotoxic lymphocytes is about 15% (unpublished data).

Recent research by other research groups has shown that T cells impair the mineralization process (25) and that terminally differentiated cytotoxic T cells (CD3+ CD8+ CD57+) in particular have a negative effect on bone regeneration in humans (26). In this study, it was demonstrated in patients with isolated closed tibial plateau fractures that the concentration of T cells (CD3+) with the phenotype CD8+CD11a++CD57+ in the circulation was increased in the group of patients with delayed bone healing and that these cells also accumulated in the fracture hematoma. Similarly, the depletion of CD8 cells in mice led to significantly improved fracture healing. The possible underlying mechanism was investigated in functional analyses. It has been shown that these cells secrete increased levels of interferon (IFN)-γ and tumor necrosis factor(TNF)-α. Both mediators, but also medium conditioned by isolated CD8+CD11a++CD57+ T cells, inhibit the osteogenic differentiation of MSC *in vitro*, which may explain the delayed bone healing *in vivo* (26). This T-cell population has also been demonstrated in rats (27).

Considering the relatively high BMC concentration in the replacement membrane used as shown in previous work done (23), the presence of CD8-positive T cells in BMC and the inhibitory effect of CD8+ cells on bone healing, it can be hypothesized that negative effects, possibly mediated by a high CD8 T cell concentration in the defect area, counteract the positive effects of the other cell populations in BMC and thus lead to a delay in bone defect healing in the rat’s femur defect model. Thus, depletion of the CD8 population from BMC could lead to a further improvement of the single-stage membrane technique.

This hypothesis was tested in an animal study using the 5 mm femoral defect model of the rat. The defects were treated with the single-stage membrane technique. The hADM, either unloaded, loaded with BMC or loaded with CD8 T-cell depleted BMC, was wrapped around the defect, which was filled with vital bone material in each group. BMC and bone material were obtained from strain-, age- and sex-matched donor rats.

## Materials and methods

### Animal selection, care and ethics

Fluctuations in hormone levels in female rats can complicate the interpretation of the data and lead to large deviations in the results. Therefore, we initially considered a stable system with male rats - as established in our group - without major hormonal fluctuations to be suitable for answering our research question (28).

Twenty-five 8-10 week old male Sprague-Dawley rats (median weight: 370 g, interquartile range=IQR 250-387 g; Janvier Labs, Saint Berthevin, France) were included in this study and housed according to the following animal welfare standards: 3 to 4 rats per cage with free access to food and water, kept in a light-controlled room (14 hours of light and 10 hours of darkness per day) with a temperature of 15 to 21°C and air circulation. The condition of the animals was checked daily by a veterinarian for one week postoperatively and weekly thereafter. The animal experiment protocol was registered by our institute (Department of Trauma Surgery and Orthopaedics, University Hospital, Goethe University Frankfurt) and approved by the Animal Care and Monitoring Committee of the Regional Council (Regierungspräsidium) in Darmstadt, Germany (Project No. FK/2020) in accordance with German law.

### Study design and groups

A total of eleven donor rat femurs were prepared for isolation of BMCs by density gradient centrifugation (29). In addition, BMCs’ CD8 cells were depleted using immunomagnetic cell sorting in one group (30). Subsequently, the human acellular dermis (hADM) was seeded with the appropriate cells and then implanted into the recipient rats prior to placement of the defect filling in the 5 mm femoral defect area.

Depending on the treatment of the hADM, the recipient rats were assigned to three groups: All groups received syngeneic spongiosa as defect filling and additionally, hADM wrapped around the defect without BMC (G1), hADM colonized with BMC (G2) and hADM colonized with BMC depleted from CD8+T-cell (G3). After eight weeks of healing, the femurs were removed for radiological, biomechanical, histological/immunohistological examination (**Table 1**).

**Table 1:** Group designations, defect filling, healing time and number of animals per group.

Group	Designation	Defect filling	Healing time	animals (n)
1	hADM	Syng. Spong.	8 weeks	9
2	hADM+BMC	-II-	-II-	8
3	hADM+BMC-CD8	-II-	-II-	8

hADM without BMC, colonized with BMC or with CD8 depleted BMC is wrapped around the 5 mm femur defect which is filled in each case with vital spongiosa from syngenic donor rats.

### Isolation of BMC and depletion of CD8-T cells

The femurs of the donor rats were removed under sterile conditions and transferred to phosphate buffered saline without Mg^2+^/Ca^2+^ (PBS-/-) supplemented with 5% penicillin/streptomycin. The proximal and distal parts of the bone were cut and the bone marrow was flushed from the bone with a syringe containing PBS-/- and 1% penicillin/streptomycin. The bone marrow was then suspended by repeated pipetting. After filtration (70 µm cell strainer, BD-Biosciences, Heidelberg, Germany), the cell suspension was washed twice, and subsequently BMCs were isolated by Ficoll density gradient centrifugation (Ficoll, 1.077g/cm^3^, Biochrom, Berlin, Germany, centrifugation: 30 min, 400 x g, 20°C). The interphase containing the mononuclear cells was collected and the cells were washed three times with PBS-/- followed by a centrifugation step of 10 min at 400 x g and 20°C [modified from (29)]. Finally, CD8^+^cells were depleted from the BMCs fraction (10^7^ cells) by immunomagnetic cell sorting according to the manufacturer’s instructions using anti-rat CD8α MicroBeads (130-090-318, Miltenyi Biotec, Bergisch Gladbach, Germany, https://www.miltenyibiotec.com/DE-en/products/cd8a-microbeads-rat.html) and LD columns (Miltenyi Biotec). Briefly, the cell pellet was diluted with 80 µl of beads buffer (PBS-/- containing 0.5% bovine serum albumin and 2 mM EDTA) and incubated with 20 µl of anti-rat CD8α Microbeads for 15 min at 4°C. The BMC suspension was then washed with 2 ml of beads buffer (centrifugation conditions: 400 x g for 10 min and RT) and resuspended with 1 ml of beads buffer. After inserting the MACS column into the magnetic stand, the cell suspension was added to the column and the flow through with BMC-CD8 cells was collected after washing three times with 1 ml of beads buffer [modified from (30)].

Depletion efficacy was assessed by flow cytometry (FACScalibur, BD Biosciences). BMC depleted from CD8 cells are referred to as BMC-CD8 in the following.

### Depletion efficacy

To prove depletion efficacy of CD8^+^ cells from the BMCs fraction, 200 µl of cell suspension (1 x 10^6^ BMC, each before and after depletion) was simultaneously stained with 5 µl of fluorochrome-conjugated monoclonal antibodies recognizing rat CD3 and CD8 proteins: CD3-APC (clone: G4.18, 17-0030-82) and CD8-PerCP™-eFluor 710 (clone: OX8, 46-0084-82, eBioscience; Thermo Fisher Scientific, Waltham, USA). Unstained cells were used as negative controls for each individual sample. After incubation for 30 min at 4°C in the dark and two washes with PBS-/- containing 1% bovine serum albumin, the cells were immediately analyzed by flow cytometry for the detection of cytotoxic T cells (CD3+CD8+) and non-cytotoxic T cells (CD3+CD8-). The lymphocytes in the BMC fraction were recorded based on their size (forward scatter: FSC) and granularity (side scatter: SSC). For this purpose, the lymphocytes were collected up to 10.000 events within a gate per measurement approach. Cells outside the gate, such as monocytes, or cell debris, were not included in the analysis. Graphical and computational evaluation Quadrant analysis was used to evaluate the dot-plot diagrams. The percentage of CD3+CD8+ T cells was calculated based on total BMC.

### Loading of the hADM with BMC or BMC-CD8

The hADM (Epiflex^®^) is produced from skin of serologically screened donors. It is manufactured using validated procedures, including decellularization, sterilization, and tissue preservation (31), and is approved as a medicinal product according §21 of the German Medicinal Products Act (approval number: 3003749.00.00).

Before the cells were seeded onto the hADM (Epiflex, thickness 0.8 mm, German Institute for Cell and Tissue replacement, DIZG, Berlin, Germany, DIZG-Catalogue-2022.pdf (osteocentre.org)), the hADM pieces (dimensions: 1 x 2 cm each) were wetted with the pore side down for 10 min in PBS (with Mg^2+^/Ca^2+^) at RT. hADM pieces were then loaded with BMC or BMC-CD8 (3 x 12.5 µl PBS containing 3.75 x 10^5^ cells each pipetted to the edges and center of the hADM) and incubated for 10 min at 37°C to achieve initial cell attachment. This volume was chosen because it was completely absorbed by the hADM. Additionally, the cell-loaded hADM was centrifuged five times at 300 x g for 1 min each. After this step, the hADM was inverted and reloaded with cells (see above), giving a total of 2.25 x 10^6^ cells per hADM piece. This was followed by 1 min of shaking on a benchtop vibrator at 130 rpm [modified from (22, 23)]. The prepared hADM was preserved in PBS with Mg2+/Ca2+ and used immediately after loading.

After each cell isolation protocol, the cells were counted with a hemocytometer (C-Chip, Neubauer Improved/DHC-N01) and adjusted for the corresponding application.

### Surgical procedure

The surgical procedure was performed as previously described (15, 22, 29). In brief, for anesthesia animals received 2 ml of Ketavet (70 mg/kg) and Rompun (10 mg/kg) intraperitoneally. Rats were placed in the lateral decubitus position after shaving and aseptic cleaning of the right hind limb. A lateral longitudinal incision was made over the femur. Fascia was cut and muscles between quadriceps and thighs were separated. A locking compression plate (Miniplate “Lockingplate LCP Compact Hand 1.5 straight”, DePuySynthes, Dubendorf, Switzerland, catalog number 036.000.038) was inserted into the anterior aspect of the femoral shaft. Four locking screws (1.3 mm; Compact Hand, DePuy-Synthes, Dubendorf, Switzerland) were used to fix the plate to the bone. The cortex was then cut with a Gigli saw (RI Systems, Davos, Switzerland) and a 5 mm femoral critical defect was created in the mid-shaft around the central hole of the plate. The hADM was carefully wrapped around the defect and the cavity was filled with approximately 100 µL vital bone chips. The hADM was closed by a suture with 5-0 vicryl. The wound was rinsed with sterile saline, sutured with 5-0 vicryl, and the superficial fascia and skin were closed with 5-0 prolene (Ethicon, Germany). Analgesia (2.6 mg/kg carprofen s.c) was maintained for seven days postoperatively and animals were monitored daily for abnormal behavior or complications. After eight weeks, the animals were sacrificed by inhalation anesthesia (isoflurane; 3.5-4% air/isoflurane mixture) followed by intracardiac injection of pentobarbital (500 mg/kg body weight). Healthy and treated femurs were harvested, wrapped in premoistened gauze, and stored at -80°C until analysis.

### Bone procedures

After thawing the femora, the screws were removed and the bone material (n=25) was preserved in 70% ethanol until further use. To maximize the use of sample material and reduce the number of animals required, each sample was analyzed in the following order: (1) radiology, (2) biomechanics and after decalcification (3) (immuno)histology (22, 29).

### Radiology: bone mineral density and callus volume

Bone mineral density and callus volume were assessed using a high-resolution *in vivo* micro-computer tomography CT (µCT) Skyscan 1176 (Bruker AXS, Karlsruhe, Germany). The region of interest was placed over the defect with the longitudinal axis of the femur perpendicular to the axis of the x-ray beam (Al 0.5 mm; Voltage: 50kV; current: 500 µA; Image average: 7; rotation rad: 180; rotation step: 0.5). The isotropic voxel size was 18 µm^3^. Two-dimensional CT images were scanned and reconstructed using snap and inverse convolution. The reconstructions were stored in 3D arrays. Bone mineral density (BMD) was evaluated using the software CT-Analyzer 1.18 (Bruker AXS, Karlsruhe, Germany). The ratio of newly formed bone volume (BV) to total defect volume (TV) was calculated using CT-analyzer 1.18 and ImageJ (https://imagej.nih.gov/ij/, accessed on December 17, 2020).

### Determination of bending stiffness

The biomechanical properties of the femoral defect site were measured using a destructive three-point bending procedure (materials testing machine: Zwicki-line Z5.0, Zwick-Roell, Ulm, Germany). Bending to failure was carried out by lowering a rod onto the femur at a constant deflection speed at a constant deflection rate of 0.1 mm/s. Load and deflection were recorded continuously. The bending stiffness (slope of the elastic deformation part of the load/deformation curve) was then determined using the Testexpert-II software (Zwick-Roell, Ulm, Germany) and compared to the healthy contralateral femur, which was also measured.

### Histological assessment of callus formation and vascularization

For histological and immunohistology stains, bones were fixed in 10% zinc formal fixative (Thermo-Electron, Pittsburgh, USA) for 20 hours, and decalcified for 14 days in 0.25 M Tris-base/10% EDTA (Sigma-Aldrich, Taufkirchen, Germany), pH 7.4. The decalcified bones were dehydrated (HistoCorePearl device, Leica Biosystem, Wetzlar, Germany), embedded in paraffin, sectioned (3 µm) and dried overnight at 37°C. To evaluate callus formation, Movat pentachrome staining of paraffin-embedded histological slides was performed using a staining kit according to the manufacturer’s instructions (Morphisto, Frankfurt, Germany). Vascularization was assessed by staining sections with mouse monoclonal antibody against rat α-smooth muscle actin (SMA) (2 µg/ml, Abcam, Cambridge, UK, clone: 1A4, cat. no. ab7817) overnight at 4°C. This was followed by incubation with a polyclonal HRP-conjugated secondary antibody to mouse IgG (1 hour/RT, Simple Stain Rat MAX PO, Nichirei Biosciences Inc., Tokyo, Japan, cat. no. 414171F) and detection of antibody-antigen reactivity using the antigen 3-amino-9-ethylcarbazole substrate (AEC, Sigma-Aldrich) for 10-20 min. Finally, the cells were counterstained with hematoxylin.

### Immunohistological assessment of CD8+cells and IFNγ

To detect immune cells in the bone defect area, sections were incubated overnight at 4°C with murine monoclonal antibody against rat CD8 (CD8α, 10 µg/ml, antibody online, clone: OX-8, cat no. ABIN1027698) or rabbit polyclonal anti-rat interferon gamma (IFNγ, 5 µg/ml, antibody online, cat no. ABIN669126). The next incubation step was performed with polyclonal HRP-conjugated secondary antibodies to mouse IgG (Simple Stain Rat MAX PO, Nichirei Biosciences Inc, cat. no. 414171F) or rabbit IgG (Simple Stain Rat MAX PO, Nichirei Biosciences Inc, cat. no. 414181F) for 1 hour at RT. The reaction was visualized as described above.

### Histomorphometric evaluation

All staining were examined by light microscopy. High-resolution images of the entire defect area were created by automatically stitching of several individual frames covering the entire defect (BZII-Analyzer software; Keyence, Neu-Isenburg, Germany). The software *ImageJ* was used to determine the area of new bone formation and IFNγ-positive area in the defect site. Subsequently, the percentages of defect area were calculated. CD8+ cells were given as mean cell number per microscopic high power field (200-fold magnification). Five non overlapping high power fields (up left, up right central, low left, low right part of the bone defect) were examined per slide. Histological analysis was performed by an independent observer blinded to the group set up.

### Statistical analysis

For continuous variables the non-parametric test (Kruskal-Wallis test) was used to compare the groups. When global p-values were p<0.05, *post-hoc* testing was performed using the Bonferroni-Holm corrected Conover-Iman-test. The difference between the weight measured before surgery and eight weeks after surgery was calculated for each group using the Wilcoxon matched-pairs test. Differences in categorical variables (e.g. rate of osseous bridging) between groups were analyzed using Fisher’s exact test. Statistical analysis was performed using Bias statistical package 11.10 software (Epsilon, Weinheim, Germany). A p-value ≤ 0.05 was considered significant.

## Results

### CD8 depletion efficiency

**Figure 1:**
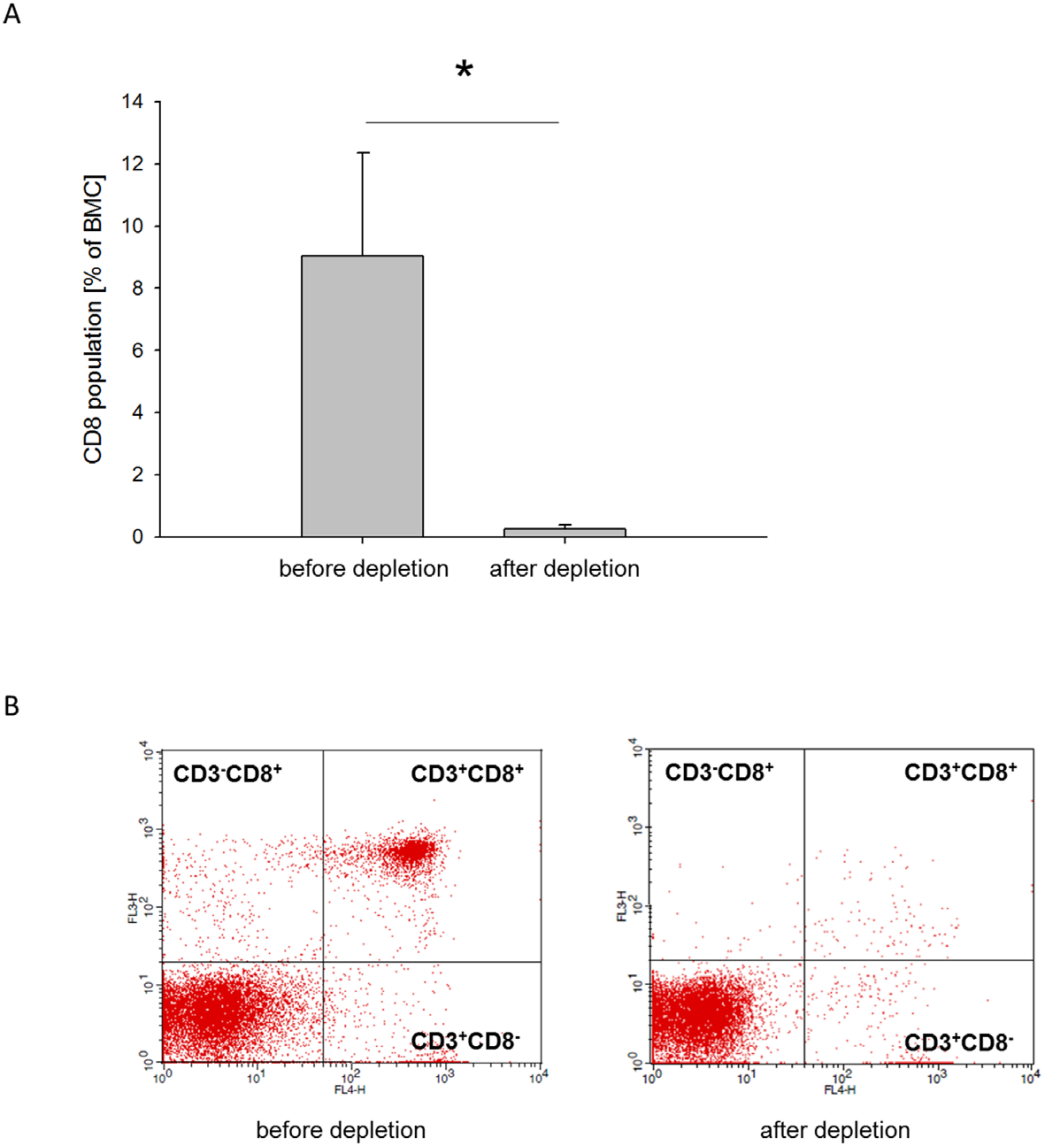
CD8 depletion efficacy. **(A)** Percentage of CD3+CD8+ cells in BMC before and after depletion of CD8+ cells (mean + standard deviation, n=3). **(B)** Representative two-color flow cytometry for CD3+CD8+ cells before CD8 depletion (left dotblot) and after CD8 depletion (right dotblot). *=p < 0.05.

To confirm the accuracy of CD8 depletion from the BMC fraction, three independent samples were isolated as described above and analyzed by flow cytometry before and after CD8 depletion. Significant differences were found between hADM+BMC+CD8 (9.05% ± 3.31) and hADM+BMC-CD8 (0.26% ± 0.12), p <0.05. The CD8-depleted samples were purified to 97% from CD8+ cells (corresponding to a 35-fold depletion, **Figure 1**).

### No differences in animal wellbeing eight weeks after surgery

No complications such as infection or pin loosening occurred during the eight weeks healing period, all animals could be included to evaluation. The median weight of the animals in each group increased significantly compared to the preoperative weight (G1: 369 g to 605 g; G2: 380 g to 600 g and G3: 370 g to 590 g), p < 0.05. There were no differences between the groups.

### Increased bending stiffness in hADM+BMC-CD8 group

**Figure 2:**
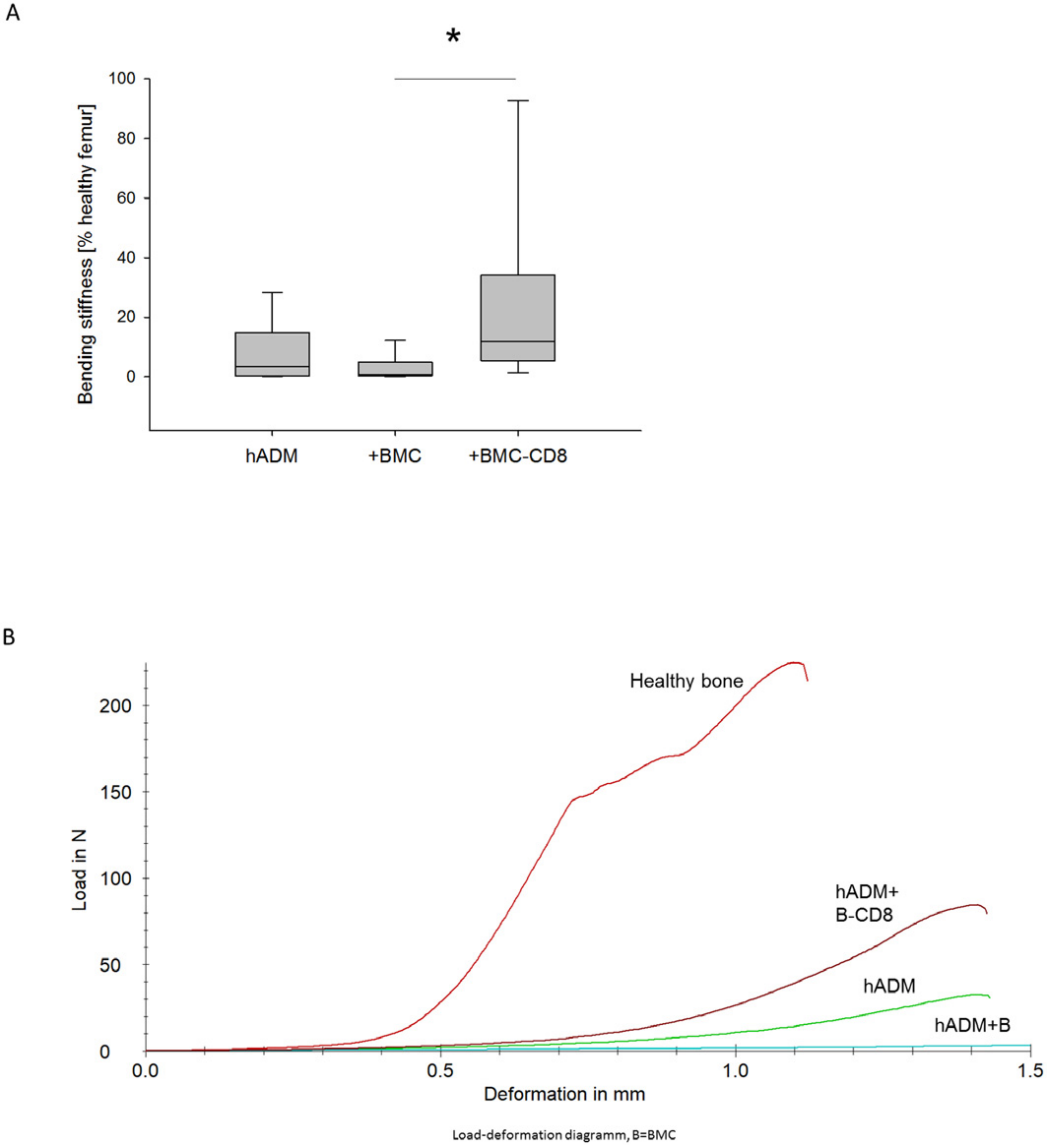
Bending stiffness of the defect zone 8 weeks after transplantation. **(A)** Bending stiffness (% healthy femur) of the defect zone **(B)** Representative force/deformation curves of a healthy femur and femurs obtained from animals receiving hADM, hADM+BMC or hADM+BMC-CD8 are shown. The peak of the curves represents the ultimate load of the bone defect. *=p < 0.05.

The bending stiffness of the defect zone was measured 8 weeks after grafting using a three-point bending test. A notable increase in femoral bending stiffness was observed in animals receiving hADM+MNC-CD8 (median: 11.7%; interquartile range=IQR 5.2-44.5) compared to the hADM+BMC group (median: 0.8%; IQR 0.17-6.7, p = 0.06), but not in comparison to the hADM group (median 3.3%: IQR 0.3-23.6, p = 0.2, **Figure 2**).

### Increased osseous bridging, BMD and BV/TV in hADM+BMC-CD8 group

**Figure 3:**
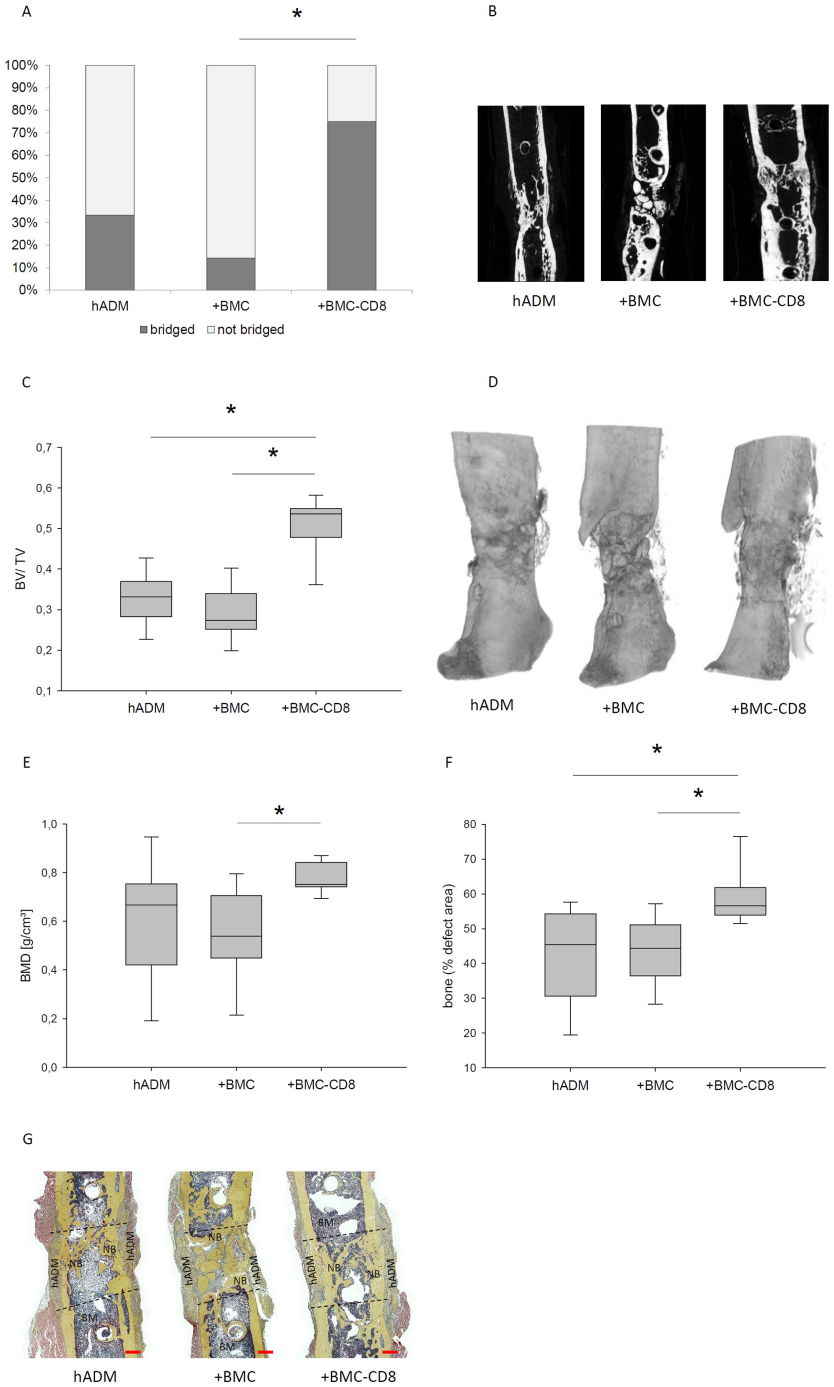
Bone healing parameters assessed by µCT and histology 8 weeks after transplantation. Proportion of defect bridging (%) and corresponding representative images are shown in **(A, B)**. BV/TV and corresponding volumen reconstructions were shown in **(C, D)**. Changes of BMD in dependency of the defect wrapping is presented in **(E)**. Histological assessment of bone formation in the defect using Movat pentachrome staining and representative histological stainings are shown in **(F, G)**. Dashed lines indicate defect borders, BM, bone marrow; NB, new bone, red bar represents 1 mm, *=p<0.05.

Defect bridging, callus volume and bone mineral density were selected as relevant factors to assess bone healing. These parameters were obtained for each sample from the respective µCT data. It was found that implantation of hADM+BMC-CD8 (n=8) into the bone defect resulted in a significantly increased ratio of osseous-bridged bone defects compared to hADM+BMC (n=7), but not compared to hADM (n=9). An increase in the BV/TV ratio was observed in animals transplanted with hADM+BMC-CD8 (n=7) compared to hADM (n=8) as well as hADM+BMC (n=8). Consistently, the hADM+BMC-CD8 group (n=7) had significantly higher BMD than the hADM+BMC+CD8 group (n=8; median 0.54 g/cm^3^, IQR 0.42-0.73), p < 0.05. However, the comparison with the hADM group (n=8) was not significant, p = 0.14. Radiological findings were generally confirmed by bone histology. Analysis of Movat’s pentachrome stained histology slides showed that bone formation was more pronounced in the defect treated with hADM+BMC-CD8 (n=8; 56%, IQR 53-62) compared to hADM+BMC (n=8; 44%, IQR 36-52) and hADM (n=9; 45%, IQR 30-54, p = <0.05, **Figure 3**).

### CD8 cell depletion has no effect on vascularization in the bone defect

The fraction of α-SMA positive area decreased in samples transplanted with hADM+BMC-CD8 (median: 0.047%; IQR 0.035 - 0.116) compared to hADM+BMC (0.101%, IQR 0.054 - 0.196) and hADM (0.077%, IQR 0.068 - 0.169), but this comparison did not reach statistical significance, p_global_= 0.26.

### Long-lasting depression of CD8+ cells after depletion

**Figure 4:**
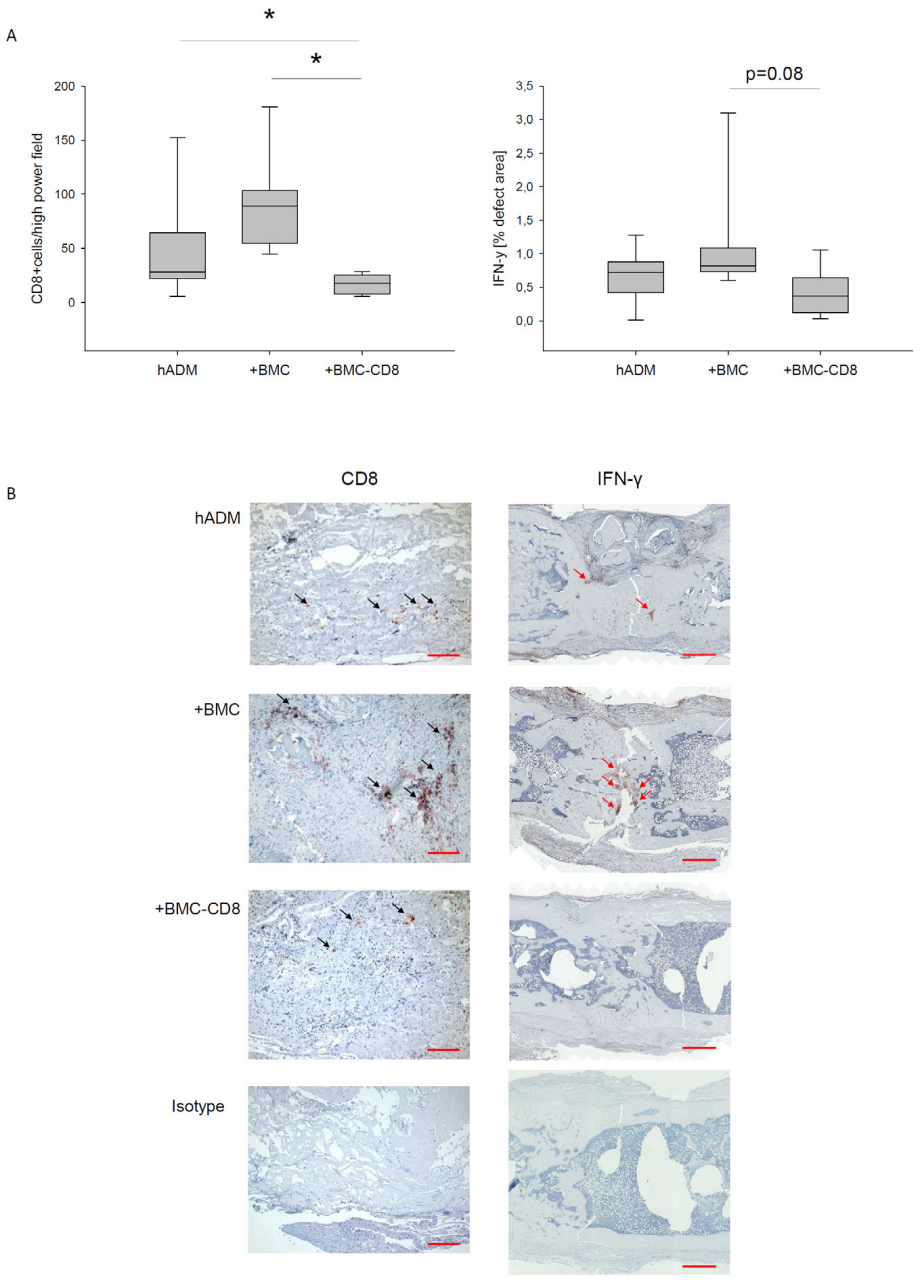
Immunohistological evaluation of CD8 and IFNγ expression. Evaluation **(A)** and representative stainings of histological sections of defects treated with hADM, hADM+BMC or hADM+BMC-CD8 **(B)** were presented. Isotype control confirmed specifity of staining. Red bar represents 100 µm (CD8) and 1 mm (IFNγ), *=p<0.05.

As expected, CD8 cell numbers in the histological sections of the callus were significantly fewer in group G3 than in groups G1 and G2, p < 0.05, respectively. Consistent with this, a particularly reduced percentage of IFNγ-positive area was observed between G3 and G2 (p=0.08). In addition, the comparison between G3 and G1 was not significant (**Figure 4**).

## Discussion

In the present study, the hypothesis was investigated whether the depletion of CD8-positive cells from BMC leads to an improvement in bone defect healing using the single-stage membrane technique. For this purpose, the hADM without cell colonization, colonized with BMC or colonized with CD8 cell-depleted BMC was placed around a 5 mm bone defect filled with vital bone material from stem matched donor rats and various bone healing parameters were analyzed after 8 weeks. It was shown that the depletion of CD8 cells led to a significant improvement in bone healing compared to complete BMC and partially compared to uncolonized hADM.

The single-stage membrane technique using a hADM represents a further development of the induced membrane technique and could be suitable for significantly reducing the treatment time for large bone defects. Using syngeneic cancellous bone as filling of the membrane pocket, an equally good bone defect healing result was achieved in the 5 mm femoral defect in rats compared to the classic two-stage induced membrane technique according to Masquelet, although the hADM is initially avital compared to the induced membrane. The concept of the single-stage membrane technique was also confirmed by Fenelon et al. also using a 5 mm femoral defect in the rat, but utilizing a human amniotic membrane (32). Generally, it is discussed that the barrier function of the membrane to the surrounding tissue plays an important role.

Nevertheless, attempts have been made to increase the efficacy of the membrane, also with regard to the use of alternative fillers of the bone defect, such as demineralized bone matrix or β-tricalcium phosphate, by colonization with BMC immediately prior to implantation.

However, these previous studies found that loading hADM with BMC resulted in a significant decrease in bone healing parameters (22, 23).

Our own analyses showed that BMC contain a significant proportion of cytotoxic T cells, and other research groups have demonstrated that cytotoxic T cells, and in particular terminally differentiated cytotoxic T cells, are associated with significantly impaired fracture healing. Further analysis has shown that this is likely due to the secretion of the potent pro-inflammatory cytokines IFN-γ and TNF-α, which inhibit, among others, the osteogenic differentiation of mesenchymal stem cells (26). The adverse effect of cytotoxic T-cells is not only limited to bone healing; clear evidence has now been found that the regeneration of other tissues, such as tendons, is also negatively influenced by these cells (33). These findings are to some extent contradictory to our own earlier studies, in which a clearly positive effect of BMC on bone defect healing was demonstrated using the rat femoral defect model (18, 19, 21). However, further studies suggested that the regenerative effect of BMC is dose-dependent and that the parameters of bone defect healing deteriorate again once a certain concentration is exceeded (34). A high BMC concentration also implies a high cytotoxic T-cell concentration. Following this line of arguments, the impaired bone healing using BMC-loaded hADM could be explained by too high BMC concentration, and thus CD8+ cell concentration. In these experiments, the BMC dose per defect was approximately 20 times higher than in the studies in which bone regenerative effects were noted with BMC loaded on bone replacement material (18).

When the CD8-positive cell population was removed from the BMCs, all measured bone healing parameters improved compared to complete BMCs. This finding impressively demonstrates the negative impact of CD8-positive T lymphocytes on the healing process. Two effects probably play a role in this. First, the presumed negative effect of CD8 T lymphocytes from the BMC preparation is greatly reduced. On the other hand, there is an increase in the absolute proportions of the remaining cell populations in the BMC preparation compared to the non-CD8-depleted BMC, since a constant number of cells was seeded in each of the BMC experimental groups. One might argue, that this lead to an increase in the absolute number of proregenerative cell populations such as monocyte/macrophages (21) which then contribute additionally to improved bone healing. The proportion of CD14-positive monocytes is approximately 14% (21). However, assuming a 10% proportion of CD8-positive cells and an equal increase of all remaining cell populations in CD8-depleted BMC, the proportion of monocytes in our experiment would increase by 10% from 14% to 15.4%. It can be assumed that this relatively small increase would not be a significant contributor to the significantly improved healing outcomes in comparison to the non-CD8 depleted group.

When CD8+T cells were depleted, at least a tendential improvement compared to the unloaded hADM could be measured for all bone healing parameters examined (bending stiffness, ratio of bridged bony defects, bone mineral density), and a significant improvement could be measured for the radiologically and histologically determined bone tissue increment. This suggests that the effectiveness of the single-stage membrane technique can be improved by additional biologization of the membrane. This also supports the hypothesis that not only the barrier function of the membrane is effective for healing, but that the biological functions such as vascularization and the release of growth factors by the cells residing within the hADM can also make a relevant contribution to bone defect healing, as is also assumed for the induced membrane according to Masquelet (35–38).

An interesting aspect is the density of CD8 cells in the defect area. The effect of depletion is clearly detectable immunohistologically even 8 weeks after surgery. The density of CD8-positive cells was significantly lower in the CD8 depletion group than in the non-depleted groups but also lower compared to the BMC-free hADM group. No clearly localized density maximum could be observed, the cells were found both in the area of the hADM and in the defect, partly locally accumulated. It has been described that cytotoxic T-lymphocytes secrete the chemokines CCL3 and CCL5, among others, and thus contribute to the auto recruitment of cytotoxic T-lymphocytes (39–42). It is conceivable that the cytotoxic T-lymphocytes introduced by BMC attract further cytotoxic T-lymphocytes of the host into the defect area via this mechanism and thus lead to a perpetuation of cytotoxic T-lymphocytes-mediated inflammation in the defect area in the sense of a positive feedback loop. This hypothesis is supported by the fact that a trend towards increased density of IFNγ-positive cells was detected immunohistologically in the non-depleted group compared to the CD8 T lymphocyte-depleted group. The increased density of IFNγ-positive cells could also be related to impaired bone healing, as IFNγ inhibits osteogenic differentiation of MSCs (25).

A previous own work suggests that the monocyte fraction in BMC is crucial for successful bone defect healing (21). It is therefore conceivable that the monocyte fraction is inhibited by the secretory activity of CD8 T-lymphocytes in their differentiation to the more regenerative M2 phenotype. Current studies show that the mediators TNFα and IFNγ, which are presumably released by CD8 T-lymphocytes, support the polarization of macrophages to the pro-inflammatory type M1 (43), which could also contribute to the maintenance of inflammation in the defect area. Thus, CD8 T-lymphocytes in our bone defect model could have a negative effect on at least two pro-regenerative processes.

An interesting aspect is the finding that an increased CD8 cell density was also recorded in the group treated with cell-free hADM, albeit lower compared to the non-depleted BMC group. We can only speculate about the reasons for this. The implanted BMC-CD8 might generate a lower chemotactic/inflammatory milieu in the membrane than cells that have entered the membrane intraoperatively by bleeding and secondarily by chemotaxis. It is conceivable that BMC-CD8 contributes to an attenuation of the pro-inflammatory cascade, which ultimately significantly influences the cellular composition in the defect area and hADM. However, the underlying immunological mechanisms would have to be analyzed in further research projects.

## Conclusion

Finally, it could be demonstrated that the single-stage membrane technique using a hADM as defect envelope can be further improved by colonization with CD8 T lymphocyte-depleted BMC. In addition, it has also been demonstrated that the fraction of CD8 T-lymphocytes in BMCs is most likely responsible for heterogeneous outcomes in BMC-assisted bone defect healing.

### Limitations

The limitation of this study is that the underlying immunological processes could not be elucidated in detail. For this purpose, experiments with a higher temporal resolution, e.g. addressing several time points in the early phase of bone defect healing, are necessary.

## Data Availability

The raw data supporting the conclusions of this article will be made available by the authors, without undue reservation.
